# The role of abnormal metabolic conditions on arterial stiffness in healthy subjects with no drug treatment

**DOI:** 10.1186/s40885-016-0049-6

**Published:** 2016-02-16

**Authors:** Hyo-Sang Hwang, Kwang-Pil Ko, Myeong Gun Kim, Sihun Kim, Jeonggeun Moon, Wook Jin Chung, Mi Seung Shin, Seung Hwan Han

**Affiliations:** From the Division of Cardiovascular Disease, Department of Internal Medicine, Gachon University Gil Hospital, Medical Center, 1198 Kuwol-dong, Namdong-gu, 405-760 Incheon, South Korea; From the Department of Preventive Medicine, Gachon University, Incheon, South Korea

**Keywords:** Cardiovascular disease, Arterial stiffness, Metabolic syndrome, Diabetes mellitus

## Abstract

**Background:**

Subjects with abnormal metabolic conditions had increased risk for cardiovascular disease. We investigated the role of abnormal metabolic conditions on arterial stiffness in healthy subjects with no drug treatment.

**Methods:**

A total 601 subjects (age 48.7 ± 11.8 years, male 46.2 %, hypertension 19.1 %) were enrolled. Major cardiovascular risk factors, metabolic conditions and parameters (pre-diabetes, diabetes mellitus, metabolic syndrome, fasting blood sugar (FBS), glycated hemoglobin), lipid profiles, hsCRP, and brachial ankle pulse wave velocity (baPWV) were measured.

**Results:**

Subjects with metabolic syndrome (*n* = 200) had higher baPWV than in subjects without metabolic syndrome (*n* = 401) even after adjustments for age, sex and presence of hypertension (1435.9 ± 212.2 vs. 1336.5 ± 225.0 cm/sec, *p* < 0.001). The differences of baPWV among normal FBS, prediabetes and diabetes mellitus groups are significant (*P* for trend = 0.036) by multivariate analysis (adjustments for age, sex, office systolic blood pressure). Subjects with diabetes mellitus (*n* = 30) had higher baPWV than in subjects with normal FBS (*n* = 384, 1525 ± 267.1 vs. 1341.5 ± 224.1 cm/sec, *P* = 0.016 adjustments for age, sex, office systolic blood pressure). BaPWV in subjects with prediabetes (*n* = 187) was slightly higher, but not statistically significant than in subjects with normal FBS (*P* = 0.377). Of interest, FBS was one of the independent predictors for increased baPWV (*β* = 0.809, 95 % CI 0.222-1.397, *p* = 0.007) by multivariate analysis.

**Conclusions:**

Subjects with abnormal metabolic conditions have increased arterial stiffness independent of age and BP which may contribute to the development of cardiovascular disease.

## Background

Pulse wave velocity (PWV), a marker of arterial stiffness, has been shown to be an independent predictor of future cardiovascular events in patients with hypertension, chronic kidney disease, coronary artery disease and even in the aged and the community [1–8]. In this regard, European Society of Hypertension/European Society of Cardiology guideline for the management of arterial hypertension recommended arterial stiffness as one of the markers for asymptomatic organ damages which can change treatment modality in hypertensive patients [9] .

Metabolic syndrome, a concurrence of disturbed glucose and insulin metabolism, overweight and abdominal fat distribution, dyslipidemia, and hypertension, is associated with subsequent development of type 2 diabetes mellitus and cardiovascular disease [10]. In addition, subjects with metabolic syndrome had increased rates of cardiovascular events and all-cause mortality [11].

In general, PWV is strongly associated with age and blood pressure (BP). However, findings with regard to its relation with other risk factors have been inconsistent and weak relation with abnormal metabolic conditions [12–17].

Because, there are still limited data for the relation between abnormal metabolic conditions and arterial stiffness and independent role of abnormal metabolic conditions on arterial stiffness, we investigate the role of abnormal metabolic conditions on arterial stiffness in healthy subjects with no drug treatment.

## Methods

### Subjects and study design

Subjects who visited for cardiovascular health examination in cardiovascular clinic were enrolled. Some of study subjects diagnosed as hypertension, diabetes mellitus and dyslipidemia. We only enrolled subjects who did not take any medications within 2 weeks of laboratory and hemodynamic measurements for more exact diagnosis of the presence of hypertension, diabetes mellitus, dyslipidemia or due to not known the presence of these conditions at visiting clinic.

The exclusion criteria for this study were subjects who already diagnosed as cardiovascular disease such as coronary artery disease, congestive heart failure, stroke, peripheral artery disease and established renal disease (serum creatinine >1.4 mg/dL), any liver disease (aspartate aminotransferase or alanine aminotransferase ≥ 3 times the upper limit of the normal in our laboratory).

Subjects were measured anthropometric data, major cardiovascular risk factors, metabolic conditions and parameters, lipid profiles, high sensitivity (hs) CRP and blood pressure (BP), heart rate and brachial ankle pulse wave velocity (baPWV). The study protocol was approved and written informed consents were waived by the local ethics committee.

### Measurements of blood pressure, heart rate and anthropometric data

Weight, height, waist and hip circumferences were measured and body mass index was calculated. Waist circumference was measured at the level of the iliac crest. Body mass index was calculated as body weight in kilograms divided by the square of height in meters (kg/m^2^). Patients underwent measurements for BP, heart rate. Before the BP measurement, patients were prohibited taking caffeine, smoking or exercise within 30 minutes. Office BP was measured from the right arm in the sitting position using automated sphygmomanometer (TM-2655P, A&D Co. Ltd., Tokyo, Japan) after at least 10 minutes of seated rest. Three measurements were taken 5 minutes apart and the mean of the last 2 values was calculated.

### Cardiovascular risk factors, lipids, metabolic conditions and parameters

Diabetes mellitus was defined as fasting glucose ≥126 mg/dL or 2 hour postprandial glucose ≥ 200 mg/dl or glycated hemoglobin (HbA1c) ≥6.5 %, or if they were already being diagnosed for this condition. Pre-diabetes was defined as fasting glucose (100 ≤ and < 126 mg/dL) or HbA1c (5.7 ≤ and <6.5). According to the revised NCEP criteria [18], an individual may be diagnosed as having metabolic syndrome if he or she has three or more of the following criteria: *1*) waist circumference >90 cm in men and >80 cm in women using the International Obesity Task Force criteria for the Asian-Pacific population to determine waist circumference [19]; *2*) triglycerides ≥150 mg/dL or medication use; *3*) HDL cholesterol <40 mg/dL in men and <50 mg/dL in women or medication use; *4*) blood pressure ≥130/85 mmHg or already being diagnosed as hypertension; and *5*) fasting glucose ≥100 mg/dL or already being diagnosed as diabetes mellitus.

Hypertension was defined as systolic BP ≥ 140 mmHg or diastolic BP ≥ 90 mmHg, or if they were already being diagnosed for this condition. Blood samples for laboratory assays were obtained following overnight fasting at least 8 hours. Total cholesterol and triglycerides were analyzed with enzymatic methods (Shinyang Chemical, Seoul, Korea), high density lipoprotein (HDL) cholesterol by a direct immunoinhibition method (Wako Pure Chemical, Osaka, Japan). LDL cholesterol was calculated using the Friedewald equation [20]. Fasting blood sugar (FBS) was determined by the hexokinase method (Shinyang Chemical, Seoul, Korea) using a Hitachi 7600–110. Assays for glycated hemoglobin (HgA1c) were measured by high performance liquid chromatography assay (VARIANT II TUR BO®, BIORAD, Inc, Hercules, California). HsCRP levels were determined with a turbidimetic assay (Denka Seiken, Tokyo, Japan) using the Hitachi 7600–110. History of smoking was obtained from all subjects.

### Measurement of brachial ankle pulse wave velocity (baPWV)

The baPWV and supine BP were measured by the automatic wave form analyzer (VP-2000, Nippon Colin Ltd, Komaki City, Japan) with simultaneous recordings of bilateral brachial and ankle BP, electrocardiogram and heart sound in a supine position after at least 5-min rest. One trained observer who did not know subject’s information performed all the measurements. The baPWV was calculated from the equation: (D1 - D2)/T. D1 is the distance between the suprasternal notch and the ankle, D2 is the distance between the suprasternal notch and the brachium, and T is the time interval between the brachium and ankle. The distances between the sampling points of baPWV are automatically calculated from the patient’s height. Pearson’s correlation coefficients of intra- and inter-observer reproducibility of baPWV were 0.976 and 0.912, respectively, as described elsewhere [21].

### Statistical analysis

Continuous variables were expressed as mean ± SD or median (25 percentile-75 percentile) and categorical variables were expressed as percentages and frequencies. Correlations between the levels of baPWV and anthropometric data, blood pressure, heart rate, laboratory data including metabolic parameters were tested using Pearson’s coefficient of correlation. To adjust the influence of age, sex, presence of hypertension or office systolic BP for baPWV, the differences of baPWV between subjects with metabolic syndrome and without metabolic syndrome and among the normal, pre-diabetes and diabetes mellitus groups were analyzed by multivariate linear regression analysis.

To determine the independent predictors for the level of baPWV, multivariate linear regression analysis was performed. All possible variables were included for this analysis if its P value of correlation co-efficiency with baPWV below 0.2. Values of *p* < 0.05 were considered significant. All tests were 2-sided.

## Results

### Study subjects and baseline characteristics

A total 601 subjects (age 48.7 ± 11.8, male 46.2 %) were enrolled. The baseline clinical characteristics are summarized in Table 1.

**Table 1:** Baseline characteristics of study subjects

Variables	Data
Age (years)	48.7 ± 11.8
Male (%)	278 (46.2)
Hypertension (%)	115 (19.1)
Pre-diabetes (%)	187 (31.1)
Diabetes mellitus (%)	30 (5)
Office systolic BP (mmHg)	129.3 ± 20.6
Office diastolic BP (mmHg)	79.0 ± 12.7
Office HR (rate)	81.0 ± 30.9
Height (cm)	163.1 ± 10.8
Weight (Kg)	65.3 ± 12.5
waist circumference (cm)	86.6 ± 9.1
hip circumference (cm)	97.0 ± 6.9
Total cholesterol (mg/dL)	203.2 ± 37.6
Triglyceride (mg/dL)	135.6 ± 4.7
HDL cholesterol (mg/dL)	52.3 ± 13.3
LDL cholesterol (mg/dL)	123.7 ± 33.7
FBS (mg/dL)	99.6 ± 22.3
HbA1c (%)	5.78 ± 0.71
hsCRP (mg/L)	0.7 (0.03-1.4)

Data are expressed as mean ± standard deviation or number (percent) or median (25 percentile −75 percentile)

*BP* blood pressure, *HR* heart rate, *HDL* high density lipoprotein, *LDL* low density lipoprotein, *FBS* fasting blood sugar, *HbA1c* glycated hemoglocinA1c, *hsCRP* high sensitivity C reactive protein

The number of subjects with hypertension was 115 (19.1 %), metabolic syndrome 200 (33.3 %). The number of subjects with normal fasting blood sugar was 384 (63.9 %), with pre-diabetes 187 (31.1 %), and with diabetes mellitus 30 (5.0 %).

### Correlations between baPWV and anthropometric data, lipids, metabolic parameters

The correlations between baPWV and anthropometric data, lipids, metabolic parameters are shown in Table 2. BaPWV are significantly correlated with age, systolic and diastolic BP. Of interest, baPWV had significant positive correlations with waist circumference, total cholesterol, triglyceride, FBS, HbA1c, creatinine and negative correlations with hip circumference, HDL-cholesterol.

**Table 2:** Correlations between baPWV and anthropometric data, lipids, metabolic parameters

Variables	r	*P*
Age	0.456	<0.001
Office systolic BP	0.426	<0.001
Office diastolic BP	0.424	<0.001
Office HR	0.048	0.241
Height	−0.68	0.094
Weight	−0.042	0.307
Waist circumference	0.132	0.002
Hip circumference	−0.086	0.040
Total cholesterol	0.112	0.006
Triglyceride	0.219	<0.001
HDL cholesterol	−0.097	0.017
LDL cholesterol	0.050	0.220
FBS	0.163	<0.001
HbA1c	0.191	<0.001
hsCRP	0.064	0.120
Creatinine	0.132	0.001

*baPWV* brachial ankle pulse wave velocity, *BP* blood pressure, *HR* heart rate, *HDL* high density lipoprotein, *LDL* low density lipoprotein, *FBS* fasting blood sugar, *HbA1c* glycated hemoglocinA1c, *hsCRP* high sensitivity C reactive protein

### Comparison of baPWV between subjects with metabolic syndrome and without metabolic syndrome

**Fig. 1:**
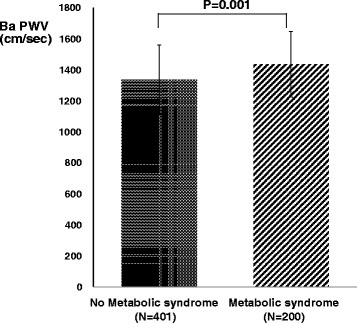
The comparison of baPWV between subjects with metabolic syndrome and without metabolic syndrome. Subjects with metabolic syndrome had significant higher baPWV than in subjects without metabolic syndrome after adjustments of age, sex, presence of hypertension by multivariate linear regression analysis. Data are presented as mean ± standard deviation of the mean. baPWV = brachial ankle pulse wave velocity

Subjects with metabolic syndrome had significant higher baPWV than in subjects without metabolic syndrome after adjustments of age, sex, presence of hypertension by multivariate linear regression analysis (1435.9 ± 212.2 vs. 1336.5 ± 225.0 cm/sec, *p* = 0.001, Fig. 1).

### Comparisons of baPWV among normal FBS, pre-diabetes and diabetes mellitus groups

**Fig. 2:**
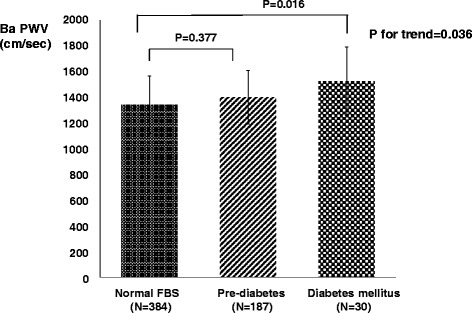
The comparisons of baPWV among normal fasting blood sugar, pre-diabetes and diabetes mellitus groups. The differences of baPWV among three groups are significant (*P* for trend = 0.036) by multivariate linear regression analysis (adjustments for age, sex, office SBP). Subjects with diabetes mellitus had higher baPWV than in subjects with normal FBS (*P* = 0.016). BaPWV in subjects with pre-diabetes is slightly higher, but not statistically significant than in subjects with normal FBS (*P* = 0.377). Data are presented as mean ± standard deviation of the mean. baPWV = brachial ankle pulse wave velocity. FBS = fasting blood sugar. SBP = systolic blood pressure

The differences of baPWV among three groups are significant (*P* for trend = 0.036) by multivariate linear regression analysis (adjustments for age, sex, office SBP, Fig. 2). Subjects with diabetes mellitus had higher baPWV than in subjects with normal FBS (1525.2 ± 267.1 vs. 1341.5 ± 224.1 cm/sec, P = 0.016). BaPWV in subjects with pre-diabetes is slightly higher (1402.4 ± 207.4 cm/sec), but not statistically significant than in subjects with normal FBS (*P* = 0.377).

### Independent factors for baPWV

The results of multivariate linear regression analysis of independent factors for baPWV are shown in Table 3. As we expected, old age and systolic, diastolic BP were significant independent predictors for increased baPWV. In addition, male sex and hip circumference were independent factors for baPWV. Of interest, greater FBS was one of the independent predictors for increased baPWV (b = 0.809, 95 % CI [0.222-1.397], *p* = 0.007).

**Table 3:** Independent factors for baPWV by multivariate linear regression analysis

	*β*	95 % CI	*P*
Age	9.696	8.513, 10.880	<0.001
Male	63.194	34.598, 91.790	<0.001
Office Systolic BP	2.771	1.558, 3.983	<0.001
Office diastolic BP	4.476	2.480, 6.472	<0.001
Hip circumference	−4.930	−6.971, −2.888	<0.001
FBS	0.809	0.222, 1.397	0.007

*baPWV* brachial ankle pulse wave velocity, *BP* blood pressure, *FBS* fasting blood sugar

## Discussion

Our study revealed that abnormal metabolic conditions such as metabolic syndrome and diabetes mellitus had increased arterial stiffness in healthy subjects with no drug treatment even after adjustments of age, sex and blood pressure. In addition, increase FBS was independent predictor for the increased arterial stiffness in the multivariate analysis. Our study demonstrated that abnormal metabolic conditions increased arterial stiffness which is one of the risk factors for cardiovascular disease.

Metabolic syndrome is associated with subsequent development of type 2 diabetes mellitus and cardiovascular disease [10]. In addition, subjects with metabolic syndrome had increased cardiovascular events and all-cause mortality [11]. Although poor clinical outcomes of metabolic syndrome are partly explained by consequence of insulin resistance and abnormal endothelial function [22–25], there are still limited data on the mechanisms of poor outcomes in subjects with metabolic syndrome for cardiovascular disease. In current out study, abnormal metabolic conditions such as metabolic syndrome and diabetes mellitus had greater arterial stiffness than in subjects with healthy metabolic state, these results suggest that increased arterial stiffness in subjects with abnormal metabolic conditions is one of the mechanisms for the poor cardiovascular outcomes in subjects with abnormal metabolic conditions.

Previous some studies reported that abnormal metabolic conditions were related with increased arterial stiffness [12–16, 26], however, there are still controversy for this topic, especially in subjects without cardiovascular disease and with no drug treatment. In one small study using MRI demonstrated that carotid pulse wave velocity was increased in middle aged subjects with metabolic syndrome, however, these results did not show in elderly subjects [26]. In large study including total 8599 subjects who underwent health examination in South China, baPWV were positively correlated with metabolic syndrome and its individual components. In this study, BP and FBS had the strongest correlation factors [14]. In another study, high BP and elevated FBS were associated with increased baPWV in patients underwent voluntary healthy checkup [16]. The results of these studies are consistent with current our study. In patients with metabolic syndrome, one rural community cohort of Korea, the ARIRANG study, revealed that arterial PWV did not show significant increase in patients with metabolic syndrome, especially in male [13]. In subjects without clinical atherosclerotic cardiovascular disease, diabetes, or systemic disease, subjects with metabolic syndrome was associated with an increased baPWV, however, none of the components of the metabolic syndrome, except for an elevated BP, was an independent factor for baPWV [27]. These results suggest that there are still needs for further investigations regarding the relationships between metabolic conditions and arterial stiffness.

In current our study, FBS is one of the independent predictors of increased arterial stiffness by multivariate analysis. In addition, our study demonstrate that abnormal metabolic conditions such as metabolic syndrome and diabetes mellitus had increased arterial stiffness even after adjustments of age, sex and blood pressure in healthy subjects with no drug treatment. Our results suggest that abnormal metabolic parameters or conditions are one of the mechanisms for increased arterial stiffness independent of age and blood pressure.

The role of hip circumference on arterial stiffness is not consistent in previous studies [28, 29]. Our study showed larger hip circumference had protective role for increased aortic stiffness.

Current our study had some limitations. Enrolled subjects were visited in cardiovascular department to check up their cardiovascular health. Therefore, the generalization of our results should be taken with caution and selection bias could exist. Second, we used baPWV instead of carotid-femoral PWV (cfPWV) to assess arterial stiffness [12]. Although cfPWV is considered the gold standard for the measurement of arterial stiffness in Western countries [30] no single methodology is proven to be superior. Regarding for efficacy for baPWV, Tanaka et al.[31] reported that the cfPWV and baPWV indices of arterial stiffness are similarly associated with cardiovascular risk factors and clinical events. Third, single measurements of variables including metabolic conditions and parameters may not represent the exact measurements of subjects’ conditions. Forth, we did not evaluate the clinical outcomes of study subjects. Further studies regarding the prognostic role of increased arterial stiffness related with abnormal metabolic conditions on cardiovascular outcomes will be required in the future.

## Conclusions

Subjects with abnormal metabolic conditions have increased arterial stiffness independent of age and BP which may contribute to the development of cardiovascular disease. In addition, increased FBS was one of the independent predictors of increased arterial stiffness. Our results suggest that treatments for abnormal metabolic conditions including lifestyle modification, drug treatments may improve arterial stiffness, which can improve subject’s cardiovascular outcomes.

## Abbreviations

baPWV: brachial ankle pulse wave velocity
BMI: body mass index
BP: blood pressure
cfPWV: carotid femoral pulse wave velocity
FBS: fasting blood sugar
HbA1c: glycated hemoglobin A1c
HDL: high density lipoprotein
hsCRP: high sensitivity C-reactive protein
LDL: low density lipoprotein

## References

[1] Laurent S, Boutouyrie P, Asmar R, Gautier I, Laloux B, Guize L (2001). Aortic stiffness is an independent predictor of all-cause and cardiovascular mortality in hypertensive patients. Hypertension.

[2] Blacher J, Pannier B, Guerin AP, Marchais SJ, Safar ME, London GM (1998). Carotid arterial stiffness as a predictor of cardiovascular and all-cause mortality in end-stage renal disease. Hypertension.

[3] Kingwell BA, Gatzka CD (2002). Arterial stiffness and prediction of cardiovascular risk. J Hypertens.

[4] Blacher J, Guerin AP, Pannier B, Marchais SJ, Safar ME, London GM (1999). Impact of aortic stiffness on survival in end-stage renal disease. Circulation.

[5] Vaccarino V, Holford TR, Krumholz HM (2000). Pulse pressure and risk for myocardial infarction and heart failure in the elderly. J Am Coll Cardiol.

[6] Domanski MJ, Sutton-Tyrrell K, Mitchell GF, Faxon DP, Pitt B, Sopko G (2001). Determinants and prognostic information provided by pulse pressure in patients with coronary artery disease undergoing revascularization. The Balloon Angioplasty Revascularization Investigation (BARI). Am J Cardiol.

[7] Mitchell GF, Hwang SJ, Vasan RS, Larson MG, Pencina MJ, Hamburg NM (2010). Arterial stiffness and cardiovascular events: the Framingham Heart Study. Circulation.

[8] Han SH, Park CG, Park SW, Shin SH, Ahn JC, Seo HS (2004). High aortic stiffness assessed by pulse wave velocity is an independent predictor of coronary artery calcification and stenosis in suspected coronary artery disease patients. Korean Circulation Journal.

[9] Mancia G, Fagard R, Narkiewicz K, Redon J, Zanchetti A, Bohm M (2013). 2013 ESH/ESC guidelines for the management of arterial hypertension: the Task Force for the Management of Arterial Hypertension of the European Society of Hypertension (ESH) and of the European Society of Cardiology (ESC). Eur Heart J.

[10] Lakka H, Laaksonen DE, Lakka TA (2002). THe metabolic syndrome and total and cardiovascular disease mortality in middle-aged men. Jama.

[11] Mottillo S, Filion KB, Genest J, Joseph L, Pilote L, Poirier P (2010). The Metabolic Syndrome and Cardiovascular Risk: A Systematic Review and Meta-Analysis. J Am Coll Cardiol.

[12] Cecelja M, Chowienczyk P (2009). Dissociation of aortic pulse wave velocity with risk factors for cardiovascular disease other than hypertension a systematic review. Hypertension.

[13] Ahn MS, Kim JY, Youn YJ, Kim SY, Koh SB, Lee K et al. Cardiovascular parameters correlated with metabolic syndrome in a rural community cohort of Korea: the ARIRANG study. J Korean Med Sci.25(7):1045–52. doi:10.3346/jkms.2010.25.7.1045 10.3346/jkms.2010.25.7.1045 PMC2890882 20592897

[14] Chen L, Zhu W, Mai L, Fang L, Ying K. The association of metabolic syndrome and its components with brachial-ankle pulse wave velocity in south China. Atherosclerosis.240(2):345–50. doi:S0021-9150(15)00191-4 [pii]. 10.1016/j.atherosclerosis.2015.03.031 10.1016/j.atherosclerosis.2015.03.031 25875386

[15] Czernichow S, Greenfield JR, Galan P, Jellouli F, Safar ME, Blacher J et al. Macrovascular and microvascular dysfunction in the metabolic syndrome. Hypertens Res.33(4):293–7. doi:hr2009228 [pii]. 10.1038/hr.2009.228 10.1038/hr.2009.228 20075933

[16] Hwang IC, Suh SY, Seo AR, Ahn HY, Yim E. Association between Metabolic Components and Subclinical Atherosclerosis in Korean Adults. Korean J Fam Med.33(4):229–36. doi:10.4082/kjfm.2012.33.4.229 10.4082/kjfm.2012.33.4.229 PMC3418342 22916325

[17] Safar ME, Thomas F, Blacher J, Nzietchueng R, Bureau J-M, Pannier B (2006). Metabolic Syndrome and Age-Related Progression of Aortic Stiffness. J Am Coll Cardiol.

[18] Grundy SM, Cleeman JI, Daniels SR, Donato KA, Eckel RH, Franklin BA (2005). Diagnosis and management of the metabolic syndrome an American Heart Association/National Heart, Lung, and Blood Institute scientific statement. Circulation.

[19] World Health Organization, et al. International Association for the Study of Obesity, International Obesity Task Force. The Asia-Pacific perspective: redefining obesity and its treatment. 2000:15–21

[20] Friedewald WT, Levy RI, Fredrickson DS (1972). Estimation of the concentration of low-density lipoprotein cholesterol in plasma, without use of the preparative ultracentrifuge. Clin Chem.

[21] Kim YK, Kim D (2005). The relation of pulse wave velocity with Framingham risk score and SCORE risk score. Korean Circulation J.

[22] Han SH, Quon MJ, Koh KK (2007). Reciprocal relationships between abnormal metabolic parameters and endothelial dysfunction. Curr Opin Lipidol.

[23] Han SH, Quon MJ, J-a K, Koh KK (2007). Adiponectin and cardiovascular disease: response to therapeutic interventions. J Am Coll Cardiol.

[24] Han SH, Sakuma I, Shin EK, Koh KK (2009). Antiatherosclerotic and anti-insulin resistance effects of adiponectin: basic and clinical studies. Prog Cardiovasc Dis.

[25] Park YM, Han SH, Seo JG, Lee S, Oh PC, Koh KK (2015). The role of insulin resistance and metabolic risk factors on culprit coronary plaque. Int J Cardiol.

[26] Blasco G, Balocco S, Puig J, Sanchez-Gonzalez J, Ricart W, Daunis-i-Estadella J et al. Carotid pulse wave velocity by magnetic resonance imaging is increased in middle-aged subjects with the metabolic syndrome. Int J Cardiovasc Imaging.31(3):603–12. doi:10.1007/s10554-014-0578-6 10.1007/s10554-014-0578-6 25425432

[27] Kim Y-K (2006). Impact of the metabolic syndrome and its components on pulse wave velocity. Korean J Intern Med.

[28] Wildman RP, Mackey RH, Bostom A, Thompson T, Sutton-Tyrrell K (2003). Measures of obesity are associated with vascular stiffness in young and older adults. Hypertension.

[29] Ko MJ, Kim MK, Shin J, Choi BY. Relations of pulse wave velocity to waist circumference independent of hip circumference. Epidemiol Health. 2010;32:e220100004. 10.4178/epih/e2010004 PMC2984863 21191457

[30] Laurent S, Cockcroft J, Van Bortel L, Boutouyrie P, Giannattasio C, Hayoz D (2006). Expert consensus document on arterial stiffness: methodological issues and clinical applications. Eur Heart J.

[31] Tanaka H, Munakata M, Kawano Y, Ohishi M, Shoji T, Sugawara J (2009). Comparison between carotid-femoral and brachial-ankle pulse wave velocity as measures of arterial stiffness. J Hypertens.

